# Predicting Severe Radiation Esophagitis in Patients With Locally Advanced Esophageal Squamous Cell Carcinoma Receiving Definitive Chemoradiotherapy: Construction and Validation of a Model Based in the Clinical and Dosimetric Parameters as Well as Inflammatory Indexes

**DOI:** 10.3389/fonc.2021.687035

**Published:** 2021-06-24

**Authors:** Yilin Yu, Hongying Zheng, Lingyun Liu, Hui Li, Qunhao Zheng, Zhiping Wang, Yahua Wu, Jiancheng Li

**Affiliations:** Department of Radiation Oncology, Fujian Medical University Cancer Hospital, Fujian Cancer Hospital, Fuzhou, China

**Keywords:** radiation esophagitis, esophageal squamous cell carcinoma, definitive chemoradiotherapy, nomogram model, inflammation index

## Abstract

**Objective:**

Radiation esophagitis (RE) is common in patients treated with radiotherapy (RT) for locally advanced esophageal squamous cell carcinoma (ESCC). We aim to construct a nomogram predicting the severe RE (grade ≥2) in patients with ESCC receiving definitive chemoradiotherapy (dCRT).

**Materials and Methods:**

Logistic regression was performed to evaluate the risk factors in predicting RE. Nomogram was built based on the multivariate analysis result. The model was validated using the area under the receiver operating curve (ROC) curve (AUC), calibration curves, and decision curve analyses (DCA). Spearman correlation analysis was used to evaluate the correlation between inflammation indexes.

**Results:**

A total of 547 patients with stage II–IVA ESCC treated with dCRT from the retrospective study were included. Two hundred and thirty-two of 547 patients (42.4%) developed grade ≥2 RE. Univariate analysis indicated that gender (p = 0.090), RT dose (p < 0.001), targeted therapy (p = 0.047), tumor thickness (p = 0.013), lymphocyte-monocyte ratio (LMR, p = 0.016), neutrophil-lymphocyte ratio (NLR, p < 0.001), and platelet-lymphocyte ratio (PLR, p < 0.001) were the significant factors for a higher incidence of RE. In multivariate analysis, RT dose [p < 0.001; odds ratio (OR), 4.680; 95% confidence interval (CI), 2.841–6.709], NLR (p < 0.001; OR, 0.384; 95% CI, 0.239–0.619), and PLR (p < 0.001; OR, 3.539; 95% CI: 2.226–5.626) were independently associated grade ≥2 RE and were involved in the nomogram. ROC curves showed the AUC of the nomogram was 0.714 (95% CI, 0.670–0.757), which was greater than each factor alone (RT dose: 0.615; NLR: 0.596; PLR: 0.590). Calibration curves showed good consistency between the actual observation and the predicted RE. DCA showed satisfactory positive net benefits of the nomogram among most threshold probabilities.

**Conclusions:**

The study demonstrated that RT dose, NLR, and PLR were independent risk factors for grade ≥2 RE in patients with locally advanced ESCC receiving dCRT. A predictive model including all these factors was built and performed better than it based on each separately. Further validation in large patient populations is still warranted.

## Introduction

Esophageal cancer (EC) is the seventh most common malignancy worldwide and the sixth leading cause of cancer-related death (1). It is usually diagnosed at an advanced stage, and only 30–40% of patients have the opportunity to surgery (2). Chemoradiation therapy is considered to be the standard treatment for unresectable, locally advanced EC (3). However, radiation esophagitis (RE) is a common acute toxic reaction for EC patients treated with radiotherapy (RT). It occurs during RT and often lasts several weeks after the completion of RT. Although the development of precision RT has reduced the incidence of RE, it remains the primary dose limiting adverse effect for EC (4).

Symptomatically, patients with RE are most typically evaluated based on clinical symptoms, including dysphagia, odynophagia, sternal, and epigastric chest pain, or all of these. In extremely rare cases, patients may develop acute or subacute esophageal perforation or bleeding. These symptoms can directly affect patients’ quality of life, and can be life-threatening. In patients with EC treated with RT alone or chemoradiotherapy (CRT), RE is a common adverse effect that affects the chance of local tumor control and the therapeutic effect and possibly resulting in a treatment break. Therefore, identifying predictors for RE will help provide the optimal treatment to an individual patient safely.

Although several studies have demonstrated predictive factors for RE, including clinical and dosimetric factors (5–9), no parameter has been typically accepted as the best predictive factor. Besides, most of these studies were based on lung cancer. To the best of our knowledge, there is no study that has evaluated the occurrence of RE that combined the clinical, dosimetric parameters and inflammation index among patients with locally advanced esophageal squamous cell carcinoma (ESCC) receiving definitive chemoradiotherapy (dCRT). In this study, we collected data of the clinical, dosimetric, and inflammatory parameters and the occurrence of RE in patients with locally advanced ESCC receiving dCRT, attempting to establish and validate a combined nomogram model predicting RE.

## Materials and Methods

### Patient Selection

We retrospectively reviewed the records of locally advanced ESCC patients treated with definitive chemoradiotherapy (dCRT) at the Fujian Provincial Cancer Hospital from January 2013 to June 2020. Patients eligible for this study included those with: (A) histopathologic proof of ESCC; (B) treatment with concurrent or sequential dCRT for locally advanced disease (stage II–IVA); (C) not operated on for medical reasons or according to the choice of the patient; (D) complete notes for the documentation of radiation esophagitis (RE); (E) Karnofsky score ≥70 points; (F) RT dose 50-70Gy (25-35 fractions in 5-7 weeks), 0-6 courses of platinum-based chemotherapy. Cases with distant metastasis or multiple primary diagnoses were excluded in the study. The blood biochemical data was collected within three days before therapy. Clinical staging was performed according to the 8th edition of TNM staging criteria for EC. Finally, 547 patients were eligible. This study was conducted in accordance with the principles of the Helsinki Declaration and approved by the Fujian Cancer Hospital Ethics Board (YKT2021-006-01).

### Radiotherapy

Intensity modulated radiation therapy (IMRT) was planned for all patients with a total dose of 50–70Gy, 25-35 fractions, 5 days per week. The treatment planning computed tomography (CT) scan was carried out with 2.5–3 mm slice thickness from neck to abdomen. RT was delivered with 6 MV X-rays linear accelerator. The gross tumor volume (GTV) and clinical tumor volume (CTV), as well as planned tumor volume (PTV), were outlined according to the standard issued by the National Comprehensive Cancer Network (NCCN). The dose and volume constraints for normal tissues were as follows: to the Bi-lung, V5 ≤ 65%, V20 ≤ 30%, average dose ≤18Gy; to the heart, V40< 40%; and to the spinal cord, <45Gy. All plans were completed in the treatment plan system (Philips Pinnacle, USA).

### Chemotherapy

All of the 547 eligible patients had received 0-6 courses of concurrent or sequential chemotherapy. The chemotherapy regimens were based on platinum, including (A) docetaxel 75 mg/m2 d1 or paclitaxel 135 mg/m2 d1 + nedaplatin 75 mg/m2 d2 or cisplatin 75 mg/m2 d2 or lobaplatin 50mg d2 or carboplatin AuC2 d2; (B) 5-fluorouracil (5-FU) 700-1000 mg/m2 d1-2 + cisplatin 75 mg/m2 d2.

### Inflammatory Index and Nutritional Index

The absolute lymphocytes count divided by the absolute monocytes count to calculate the lymphocyte-monocyte ratio (LMR). The absolute neutrophils count divided by the absolute lymphocytes count to calculate the neutrophil-lymphocyte ratio (NLR). The absolute platelets count divided by the absolute lymphocytes count was used to calculate the platelet-lymphocyte ratio (PLR), and the absolute platelets count multiplied by NLR to calculate the systemic immune-inflammation index (SII). Finally, the serum albumin level (g/L) + 5 multiplied by the absolute lymphocytes count to calculate the prognostic nutrition index (PNI).

### Follow-Up and Esophagitis Scoring

Radiation esophagitis was scored according to the Radiation Therapy Oncology Group (RTOG)/European Organization for Research and Treatment of Cancer radiation morbidity score system (EORTC) from the start of IMRT until three months afterward. During IMRT, patients are assessed weekly for side effects and more frequently if interventions were needed. After IMRT, follow-up monitoring was once a month. The side effect was recorded as a maximum grade at any time during treatment or follow-up period. For the purpose of our analysis, only RE greater than or equal to grade 2 was considered events. The diagnosis of RE was confirmed by two experienced radiation oncologists based on clinical symptoms and changes in CT images. The clinical RE grades were available for all patients.

### Statistical Analyses

All statistical analyses were conducted using R software (version 4.0.2) and SPSS (version 26.0). The optimal cutoff values of RT dose, tumor length, tumor thickness, PNI, LMR, NLR, PLR, and SII were calculated individually according to the receiver operating characteristics (ROC) curve or average value to select the most relevant threshold to predict RE. Logistic regression was used for univariate and multivariable analysis of the influence on the occurrence of RE. Those factors with p < 0.20 in the univariate analysis were then incorporated into the multivariate analysis to identify independent predictors of RE. Factors with significant value in multivariate analysis were performed to construct the nomogram. The validation of the nomogram was performed using the area under the ROC curve, calibration curve (with 1000 bootstrap resamples), and decision curve analysis (DCA). The ROC curves were used to evaluate the discrimination ability of each predictor alone and the nomogram. The calibration curve was performed to compare the observed probability with the predicted probability of RE. DCA was used to demonstrate the clinical validity of the nomogram by quantifying the net benefits at different threshold probabilities. Finally, Spearman correlation analysis estimated the correlation between NLR, PLR, LMR, and SII. All tests were two-tailed. A value of p < 0.05 was considered statistically significant.

## Results

### Patient Characteristics

A summary of the clinical, dosimetric, and inflammatory variables of the 547 patients with locally advanced ESCC treated with dCRT are shown in **Table 1**. Patients’ gender, age, weight loss, tumor location, RT dose, chemotherapy situation, tumor length, tumor thickness, tumor stage, PNI, LMR, NLR, PLR, and SII were collected. A total of 382 male patients and 165 female patients were analyzed in our study. The median age at diagnosis was 67 years. Of 547 patients treated with dCRT, 70.2% received a platinum-containing regimen. The majority of patients (76.4%) had Stage III/IVA disease. The optimal cutoff value for RT dose, tumor length, tumor thickness, PNI, LMR, NLR, PLR, and SII was calculated to be 61.5Gy, 5.5cm, 1.5cm, 47, 10.8, 1.71, 136.3, and 633.9, respectively.

**Table 1:** Characteristics of locally advanced ESCC patients enrolled in this study (n = 547).

Clinicopathologic variable		Total (N)	Percentage (%)
Gender			
	Male	382	69.8%
	Female	165	30.2%
Age (years)			
	<65	236	43.1%
	≥65	311	56.9%
Weight loss			
	Yes	255	46.6%
	No	292	53.4%
Tumor location			
	Cervical	43	7.9%
	Upper thoracic	152	27.8%
	Middle thoracic	285	52.1%
	Lower thoracic	67	12.2%
RT dose (Gy)			
	≤61.5	438	80.1%
	>61.5	109	19.9%
Chemotherapy			
	Yes	384	70.2%
	No	163	29.8%
Targeted therapy			
	Yes	44	8.0%
	No	503	92.0%
Tumor length (cm)			
	<5.5	316	57.8%
	≥5.5	231	42.2%
Tumor thickness (cm)			
	<1.5	241	44.1%
	≥1.5	306	55.9%
T stage			
	T2	32	5.9%
	T3	294	53.7%
	T4	221	40.4%
N stage			
	N0	165	30.2%
	N1	207	37.8%
	N2	140	25.6%
	N3	35	6.4%
TNM stage			
	Stage II	129	23.6%
	Stage III	173	31.6%
	Stage IV	245	44.8%
PNI			
	<47	280	51.2%
	≥47	267	48.8%
LMR			
	<10.8	114	20.8%
	≥10.8	433	79.2%
NLR			
	<1.71	173	31.6%
	≥1.71	374	68.4%
PLR			
	<136.3	283	51.7%
	≥136.3	264	48.3%
SII			
	<633.9	359	65.6%
	≥633.9	188	34.4%
RE			
	grade0	74	13.5%
	grade1	241	44.1%
	grade2	172	31.4%
	grade3	51	9.3%
	grade4	9	1.6%

ESCC, esophageal squamous cell carcinoma; RT, radiotherapy; PNI, prognostic-nutrition index; LMR, lymphocyte-monocyte ratio; NLR, neutrophil-lymphocyte ratio; PLR, platelets-lymphocyte ratio; SII, systemic immune-inflammation index; RE, radiation esophagitis.

### Toxicity

As this study analysis suggests, of 547 patients treated with IMRT, 232 (42.4%) patients developed grade≥2 RE, including 172 (31.4%) patients with grade 2 esophagitis, 51 (9.3%) with grade 3 and 9 (1.6%) with grade 4. RE was scored as grade 0 in 74 (13.5%) patients, as grade 1 in 241 (44.1%) patients.

### Univariate and Multivariate Survival Analysis of RE in ESCC

Univariate analysis indicated that gender (p = 0.090; OR, 0.727; 95% CI, 0.504–1.051), RT dose (p < 0.001; OR, 4.393; 95% CI, 2.783–6.394), targeted therapy (p = 0.047; OR, 1.882; 95% CI, 1.010–3.506), tumor thickness (p = 0.013; OR, 0.647; 95% CI, 0.458–0.914), LMR (p = 0.016; OR, 0.586; 95% CI, 0.379–0.906), NLR (p < 0.001; OR, 0.413; 95% CI, 0.285–0.596), and PLR (p < 0.001; OR, 2.070; 95% CI, 1.466–2.921) were the significant factors for a higher incidence of RE (**Table 2**). Those factors with p < 0.20 in the univariate analysis were then involved into the multivariate analysis. Thus, the multivariate analysis was conducted on factors including gender, weight loss, RT dose, targeted therapy, tumor thickness, tumor stage, LMR, NLR, PLR, and SII. In multivariate analysis, RT dose (p < 0.001; OR, 4.680; 95% CI, 2.841–6.709), NLR (p < 0.001; OR, 0.384; 95% CI, 0.239–0.619), and PLR (p < 0.001; OR, 3.539; 95% CI: 2.226–5.626) were independent prognosticators of RE. These factors were then used in the nomogram building.

**Table 2:** Univariate and multivariate analysis of the clinical, dosimetric factors and inflammation indexes in predicting grade ≥2 RE.

Clinicopathologic Parameters	Univariate Analysis			Multivariate Analysis		
	OR	95% CI	P	OR	95% CI	P
Gender						
Male *vs.* Female	0.727	0.504-1.051	0.090	0.717	0.474-1.083	0.114
Age (years)						
≥65 *vs.* <65	0.944	0.670-1.329	0.739			
Weight loss						
Yes *vs.* No	1.266	0.901–1.779	0.174	1.31	0.889-1.929	0.172
Tumor location						
Cervical/Upper *vs.* Middle/Lower	1.188	0.835–1.691	0.339			
RT dose (Gy)						
>61.5 *vs.* ≤61.5	4.393	2.783-6.934	<0.001	4.68	2.841-6.709	<0.001
Chemotherapy						
Yes *vs.* No	1.203	0.828-1.748	0.332			
Targeted therapy						
Yes *vs.* No	1.882	1.010-3.506	0.047	1.943	0.964-3.914	0.063
Tumor length (cm)						
≥5.5 *vs.* <5.5	0.971	0.688-1.368	0.864			
Tumor thickness (cm)						
≥1.5 *vs.* <1.5	0.647	0.458-0.914	0.013	0.69	0.464-1.026	0.067
T stage						
T3/T4 *vs.* T2	1.212	0.592-2.479	0.599			
N stage						
N1/N2/N3 *vs.* N0	0.808	0.560-1.168	0.257			
TNM stage						
Stage III/Stage IV *vs.* Stage II	0.711	0.478-1.057	0.092	0.662	0.416-1.051	0.080
PNI						
≥47 *vs.* <47	1.188	0.846-1.669	0.319			
LMR						
≥10.8 *vs.* <10.8	0.586	0.379-0.906	0.016	0.679	0.417-1.105	0.119
NLR						
≥1.71 *vs.* <1.71	0.413	0.285-0.596	<0.001	0.384	0.239-0.619	<0.001
PLR						
≥136.3 *vs.* <136.3	2.07	1.466-2.921	<0.001	3.539	2.226-5.626	<0.001
SII						
≥633.9 *vs.* <633.9	0.772	0.538-1.107	0.159	0.683	0.406-1.149	0.151

RE, radiation esophagitis; OR, odds ratio; 95% CI, 95% confidence interval; RT, radiotherapy; PNI, prognostic nutrition index; LMR, lymphocyte-monocyte ratio; NLR, neutrophil-lymphocyte ratio; PLR, platelet-lymphocyte ratio; SII, systemic immune-inflammation index.

### Development and Validation of the Nomogram

**Figure 1:**
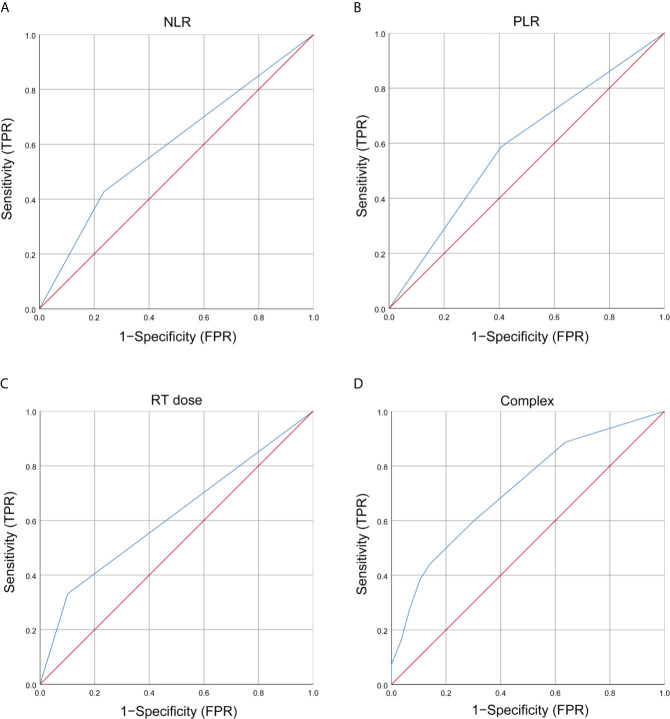
Receiver operating characteristic (ROC) curves of clinical and dosimetric factors, inflammation index, and complex for grade ≥2 RE. **(A)** ROC curves of NLR; **(B)** ROC curves of PLR; **(C)** ROC curves of RT dose; **(D)** T ROC curves of complex (NLR, PLR, and RT dose). RT, radiotherapy; NLR, neutrophils-lymphocyte ratio; PLR, platelets-lymphocytes ratio; RE, radiation esophagitis; FPR, false positive rate; TPR, true positive rate.

**Figure 2:**
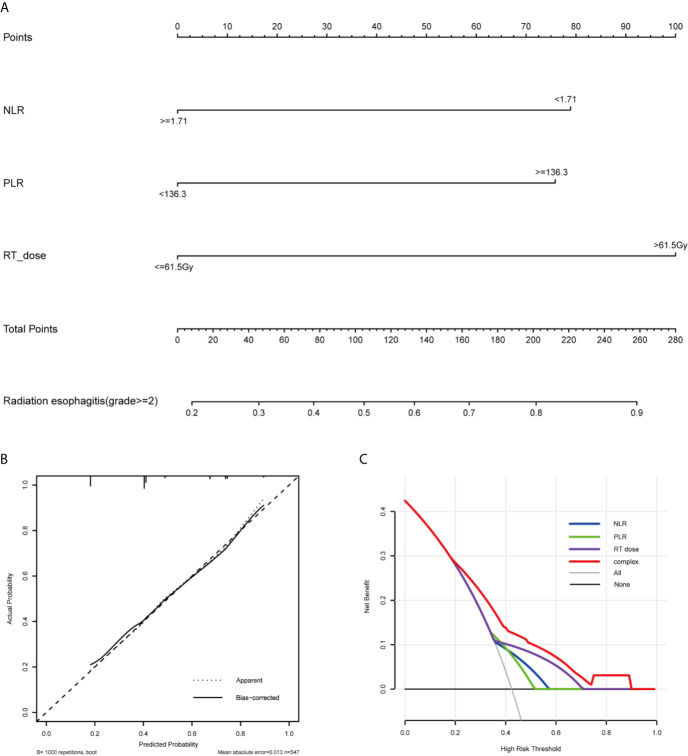
Nomogram, calibration curve, and Decision curve for predicting the probability of grade ≥2 RE for the whole study population. **(A)** A nomogram that integrates RT dose, NLR, and PLR in ESCC patients. **(B)** The calibration curve of the nomogram predicting the occurrence of grade ≥2 RE. The x-axis and y-axis indicate the predicted and actual probabilities of having grade ≥2 RE, respectively. A 45° line represented optimal predictive values. **(C)** The decision curves of the nomogram predicting the occurrence of grade ≥2 RE. The x-axis shows the threshold probabilities. The y-axis measures the net benefit, which is calculated by subtracting the false positives and adding the true positives. The horizontal line along the x-axis assumes that no patient will have grade ≥2 RE whereas the solid gray line assumes that all patients will have grade ≥2 RE at a specific threshold probability. The blue, green, purple and red line represents the net benefit of using the NLR, PLR, RT dose, and complex, respectively. RE, radiation esophagitis; RT, radiotherapy; NLR, neutrophils-lymphocyte ratio; PLR, platelets-lymphocytes ratio.

Based on the multivariate analysis result, a prediction model was presented as a nomogram. The ROC curves of NLR, PLR, RT dose, and the complex (NLR, PLR, and RT dose) were shown in **Figure 1**. The prediction model showed a good AUC of 0.714 (95% CI, 0.670–0.757) by ROC, which was much higher than individual parameter alone [NLR: 0.596, 95% CI: 0.547–0.644; PLR: 0.590, 95% CI: 0.542–0.638; RT dose: 0.615, 95% CI: 0.566–0.664 (**Figure 2A**)]. The optimal threshold values of NLR, PLR, and RT dose were <1.71, ≥136.3, and >61.5Gy, respectively. The calibration curve demonstrated favorable consistency between the actual observation and the predicted RE (**Figure 2B**). Finally, the DCA showed a satisfactory positive net benefit of the nomogram among most threshold probabilities, indicating a potential clinical effect of the nomogram model (**Figure 2C**).

### Correlation Between Inflammation Indexes

**Figure 3:**
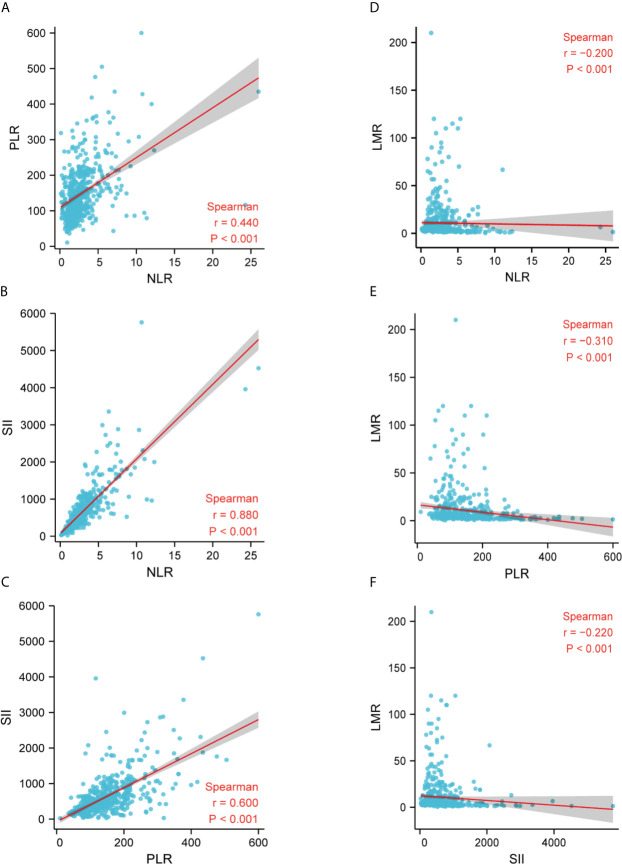
Correlation between LMR, NLR, PLR, and SII in the whole study population. **(A)** The correlation between NLR and PLR; **(B)** The correlation between NLR and SII; **(C)** The correlation between PLR and SII; **(D)** The correlation between NLR and LMR; **(E)** The correlation between PLR and LMR; **(F)** The correlation between SII and LMR. LMR, lymphocytes-monocytes ratio; NLR, neutrophils-lymphocyte ratio; PLR, platelets-lymphocytes ratio; SII, systemic immune-inflammation index; r, correlation.

We further applied Spearman correlation to analyze the correlation between LMR, NLR, PLR, and SII. Spearman’s analyses indicated a relatively strong positive correlation between NLR and PLR (r = 0.440, p < 0.001), NLR and SII (r = 0.880, p < 0.001), PLR and SII (r = 0.600, p < 0.001) (**Figures 3A–C**). Finally, we observe that LMR is negatively correlated with NLR (r = -0.200, p < 0.001), PLR (r = -0.310, p < 0.001), and SII (r = -0.220, p < 0.001), respectively (**Figures 3D–F**).

## Discussion

Definitive chemoradiotherapy (dCRT) acts as an irreplaceable role in management of advanced esophageal cancer (EC) (10). It is suitable for certain subgroups of some patients, especially in those with cT4, extensive lymph node metastasis, or unable to operate. It has been proved to be as effective as surgery in patients with stage II to IV (11). For patients who are unsuitable for surgery or show a favored response to chemoradiotherapy, dCRT may be better for surgery (12, 13). However, Study has shown that patients with esophageal squamous cell carcinoma (ESCC) have a higher local recurrence rate than esophageal adenocarcinoma (EAD) (14). Therefore, a higher dose of RT might be necessary (15–18). However, once the RT dose is increased, the chance of severe radiation esophagitis (RE) will increase, which will affect the quality of life and treatment efficacy of patients. Therefore, it is crucial to recognize some factors related to severe RE.

In the present study, we built a multivariate model for grade ≥2 RE in patients with locally advanced ESCC treated with dCRT. We investigated the effect of gender, age, weight loss, tumor location, RT dose, chemotherapy situation, tumor length, tumor thickness, tumor stage, PNI, LMR, NLR, PLR, and SII on the risk of grade ≥2 RE. Our data indicated that RT dose, NLR, and PLR were significantly associated with grade ≥2 RE. To the best of our knowledge, it is the first nomogram model to evaluate the occurrence of RE that combined RT dose, NLR, and PLR in locally advanced ESCC patients treated with dCRT ever reported. The model showed significant favorable agreement and discriminative ability between the observed outcome and predicted risk. Bootstrap validation demonstrated the robustness of the model to future similar populations. The decision curve analysis (DCA) also indicated potential clinical effects for future treatment and plan evaluation in clinical practice. The internal validation of the model indicated its advantage compared with any single clinical and dosimetric factor alone.

There is some literature confirming the relationship between RE and radiation dose (7, 9). Identically, in our univariate analysis result, there was a correlation between grade ≥2 RE and RT dose. For patients with locally advanced ESCC treated with dCRT, the recommended dose for radiation therapy is 50-70Gy. However, there is still no uniform principle for evidence-based medicine for RT dose. In the present study, our results indicated that patients receiving RT dose >61.5Gy were more likely to develop grade ≥2 RE than those receiving RT dose ≤61.5Gy. We suggested that a more comprehensive therapeutic schedule for patients treated with dCRT should be considered to reduce or avoid therapeutic toxicity. We demonstrated that the esophageal dosimetric parameter was an essential factor for predicting RE, and controlling the esophageal RT dose is a crucial step in reducing the risk of RE.

In recent years, some biological factors have been investigated as predictors of the occurrence of RE. Several studies have shown that patients receiving hematopoietic growth factors or exogenous cytokines have an increased incidence of RE and radiation pneumonia in lung cancer patients (19–22). One study has also linked inflammatory markers and RE in lung cancer patients. Guerra et al. indicated that inflammatory markers are related to RE (23). These studies emphasize the importance of inflammation in RT toxicity. However, there is no literature about the role of baseline inflammation index and the incidence of RE in locally advanced ESCC patients receiving dCRT. As far as I know, it is the first study to evaluate the occurrence of RE and inflammation index in locally advanced ESCC patients. According to our univariate and multivariate analysis results, NLR and PLR was independently factor of the RE grade ≥2. In our study, NLR <1.71 and PLR ≥136.3 showed significant associations with grade ≥2 RE occurrence in this study. Interestingly, this result was consistent with the other studies in patients with lung cancer receiving dCRT. Tang et al. indicated that pretreatment hemogram changes, specifically higher platelets and lower hemoglobin, may be more sensitive to grade ≥2 RE (24). De Ruysscher et al. showed an association about neutropenia during CRT and RE (25, 26). Decreased neutrophil count and increased platelet count reflect the reaction of the bone marrow to the organism’s systemic inflammation (27). Indeed, having a lower neutrophil count and higher platelet count may reflect a systemic inflammatory state, which when combined with CRT induced inflammation, results in RE.

Though it is generally believed that the predictive model based on clinical and dosimetric parameters has favorable discriminative ability, additional biomarkers help to improve the predictive ability of RE. De Ruyck et al. constructed a model that involves clinical, dosimetric, and genetic parameters that were found to be highly predictive for the incidence of grade ≥2 RE in patients with lung cancer. The sensitivity of the model is 84.0%, and the specificity is 75.3% (28). From the perspective of individual medicine, a more accurate prediction model combining the clinical, dosimetric, and genomic parameters should be developed in the future. More works need to be done to find easily available and clinically useful biomarkers.

Our study had several limitations. Firstly, the potential selection bias existed in retrospective analyses. Secondly, our study is limited to patients with ESCC and has no guiding significance for patients with EAD. Thirdly, there may be some confounding factors that are not included in our study. In addition, due to the limitations of endpoint events and retrospective study, we did not perform the analyze of grade ≥3 RE and grade ≥2 late toxicity in our study. Finally, the study was designed in a relatively homogeneous group of patients receiving treatment in a single institution. It is necessary to verify the results by an external validation in future studies.

## Conclusions

In conclusion, the study demonstrated that RT dose, NLR, and PLR were independent risk factors for grade 2 or higher RE in patients with locally advanced ESCC receiving dCRT. A predictive model including all these factors was built and performed better than it based on each separately. Further validation in large patient populations is still warranted.

## Data Availability Statement

The raw data supporting the conclusions of this article will be made available by the authors, without undue reservation.

## Ethics Statement

The current study was approved by the ethics committee of Fujian Medical University Cancer Hospital, Fuzhou, China (YKT2021-006-01) and conducted in accordance with the principles of the Declaration of Helsinki and its amendment. All patients provided written informed consent prior to treatment, and all the information was anonymized prior to analysis.

## Author Contributions 

JL, YY, and HZ designed this study. LL and HL contributed to the data collection. YY, HZ, and QZ analyzed the data. JL supervised the study. YY, ZW and YW wrote the manuscript. All authors contributed to the article and approved the submitted version.

## Funding

This work was supported by the Joint Funds for the Financial Foundation of Fujian Province [Grant number: (2020)729].

## Conflict of Interest

The authors declare that the research was conducted in the absence of any commercial or financial relationships that could be construed as a potential conflict of interest.

## Abbreviations

RE, radiation esophagitis; ESCC, esophageal squamous cell carcinoma; dCRT, definitive chemoradiotherapy; ROC, receiver operating curve; AUC, the area under the receiver operating curve; DCA, decision curve analyses; RT, radiotherapy; LMR, lymphocyte-monocyte ratio; NLR, neutrophil-lymphocyte ratio; PLR, platelet-lymphocyte ratio; OR, odds ratio; CI, confidence interval; EC, esophageal carcinoma; CRT, chemoradiotherapy; IMRT, intensity modulated radiation therapy; CT, computed tomography; GTV, gross tumor volume; CTV, clinical tumor volume; PTV, planned tumor volume; NCCN, National Comprehensive Cancer Network; SII, systemic immune-inflammation index; PNI, prognostic nutrition index; RTOG, Radiation Therapy Oncology Group; EORTC, European Organization for Research and Treatment of Cancer radiation morbidity score system; EAD, esophageal adenocarcinoma; FPR, false positive rate; TPR, true positive rate; r, correlation.
